# Predictors of Secondary Pulmonary Hypertension-Related Hospitalizations and Subsequent Mortality in Adults with Obstructive Sleep Apnea

**DOI:** 10.3390/diseases14020073

**Published:** 2026-02-16

**Authors:** Hassaan Imtiaz, Adil Sarvar Mohammed, Avilash Mondal, Lakshmi Sai Meghana Kodali, Sai Gautham Kanagala, Rupak Desai, Umera Yasmeen, Haritha Darapaneni, Muhammad Usman Ghani, Shweta Kambali, Shrinivas Kambali, Mohd S. Kanjwal

**Affiliations:** 1 Department of Internal Medicine, McLaren Bay Region, Bay City, MI 48708, USA; hassaan.imtiaz214@gmail.com (H.I.); shweta.kambali@mclaren.org (S.K.); 2 Department of Internal Medicine, Central Michigan University, Saginaw, MI 48602, USA; harit1d@cmich.edu (H.D.); ghani1mu@cmich.edu (M.U.G.); 3 Division of Cardiovascular Medicine, West Virginia University, Morgantown, WV 26506, USA; avilashmandal98@gmail.com; 4 Department of Public Health & Health Sciences, University of Michigan–Flint, Flint, MI 48502, USA; meghanakodali01@gmail.com; 5 Department of Internal Medicine, New York Medical College, Metropolitan Hospital Center, New York, NY 10029, USA; gauthamkanagala@gmail.com; 6 Independent Outcomes Researcher, Atlanta, GA 30033, USA; drrupakdesai@gmail.com; 7 Department of Internal Medicine, Mamata Medical College, Khammam 507002, Telangana, India; umera.uy@gmail.com; 8 Department of Pulmonary and Critical Care Medicine, MyMichigan Medical Center, Saginaw, MI 48601, USA; shrikamb81@gmail.com; 9 Department of Pulmonary and Critical Care Medicine, Covenant HealthCare, Saginaw, MI 48602, USA; mskanjwal@yahoo.com

**Keywords:** obstructive sleep apnea, secondary pulmonary hypertension, inpatient mortality, gender disparities, racial disparities

## Abstract

Background: Secondary pulmonary hypertension (SPH) predicts poor outcomes in obstructive sleep apnea (OSA) patients. This study examines sex/racial disparities, predictors, and inpatient mortality in SPH-related OSA hospitalizations. Methods: We used the National Inpatient Sample (2019) and ICD-10 codes to identify OSA-related hospitalizations with SPH. The burden of SPH and disparities by sex/race were assessed. We also compared the odds and predictors of in-hospital mortality in OSA patients with vs. without SPH. Results: Of total adult OSA hospitalizations (*n* = 2,317,136, median age of 66 [56–74] years, and males: 57.2%), 9.4% (218,795/2,317,136) had SPH. Females vs. males (11.3% vs. 8.1%) and Blacks vs. other race groups (13.5%) with OSA had a higher prevalence of SPH. The SPH cohort often consisted of females (51 vs. 41.9%), Blacks (20.9 vs. 14.0%), Medicare-insured (73.4 vs. 60.6%), and non-elective admissions (89.2 vs. 74.4%) vs. the non-SPH cohort. The SPH cohort also had a higher burden of complicated HTN (52.9 vs. 36.3%), DM with complications (42.7 vs. 32.4%), COPD (52.5 vs. 36.9%), history of prior MI (11.4 vs. 9.6%), and venous thromboembolism (10.4 vs. 8.4%). However, in-hospital mortality was more likely to be in males (OR 1.12; 95%CI 1.00–1.25, *p* = 0.048) vs. females, and OSA patients with metastatic cancer (OR 2.73; 95%CI 2.04–3.65) and solid non-metastatic tumors (OR 1.65; 95%CI 1.26–2.15) (*p* < 0.001). Conclusions: The prevalence of SPH with OSA was greater in females and Blacks, whereas males and Whites had higher subsequent inpatient mortality. More prospective studies are needed to understand the role of comorbidities on survival outcomes.

## 1. Introduction

Pulmonary hypertension (PH) is defined as mean pulmonary arterial pressure (mPAP) greater than 25 mmHg measured using right heart catheterization. It is subclassified as pre-capillary (pulmonary arterial wedge pressure) (PAWP ≤ 15 mm Hg) and post-capillary (PAWP > 15 mm Hg) based on hemodynamic parameters [1]. Pulmonary arterial hypertension, pulmonary diseases, and chronic thromboembolic phenomenon comprise pre-capillary causes, while cardiac conditions usually lead to post-capillary causes of PH. Sleep disorder breathing is hypothesized to have its deleterious effects on the pre-capillary portion of the pulmonary circuit [2].

One such example of sleep disordered breathing would be obstructive sleep apnea (OSA), which is characterized by episodes of intermittent hypoxemia and sleep fragmentation. It is one of the most prevalent comorbid conditions, especially in patients with cardiac risk factors such as hypertension, heart failure, coronary artery disease, and pulmonary hypertension, with its prevalence being as high as 40% to 80% in these patients [3,4]. OSA is prevalent in almost two-thirds of patients diagnosed with PH [5]. OSA causes hypoxic pulmonary vasoconstriction, and when combined with pulmonary venous hypertension secondary to increased left-sided pressures from heart failure, a common comorbid condition in these patients, it leads to pulmonary hypertension [6]. This is explained by the abnormal production of mediators resulting in vascular cell proliferation and aberrant vascular remodeling. A recent study from the Pulmonary Hypertension Association Registry (PHAR) concluded that the long-term mortality and morbidity in PH patients can be anywhere from 20% to 55% after 3 years [7]. Despite growing recognition of the association between OSA and SPH, large-scale data examining demographic disparities, comorbidity-specific predictors, and short-term outcomes of SPH-related hospitalizations in OSA patients remain limited. Prior studies have often been constrained by small sample sizes, referral bias, or lack of national representation, limiting their generalizability. Furthermore, the intersection of sex, race, and comorbidity burden in shaping SPH risk and inpatient outcomes among OSA patients is not well characterized.

Hence, it is imperative to understand the prevalence, predictors, and outcomes in patients with sleep disorders who have a high risk of PH. Therefore, using a nationally representative administrative dataset, we conducted an exploratory, hypothesis-generating analysis to identify demographic characteristics, clinical comorbidities, and hospital-level factors associated with SPH-related hospitalizations and subsequent in-hospital mortality among adults with OSA.

Study Aims:

(1) To describe demographic characteristics, comorbidity burden, and unadjusted in-hospital outcomes and resource utilization among OSA hospitalizations with and without SPH.
(2) To identify independent predictors of SPH-related hospitalization among adults hospitalized with OSA.
(3) To identify independent predictors of all-cause in-hospital mortality among OSA hospitalizations with SPH.

## 2. Methods

We performed a retrospective cohort study of hospitalizations during calendar year 2019 using the National Inpatient Sample (NIS), a component of the Healthcare Cost and Utilization Project sponsored by the Agency for Healthcare Research and Quality [8]. The NIS is derived from State Inpatient Databases and approximates a 20% stratified sample of discharges from U.S. community hospitals, excluding rehabilitation and long-term acute care facilities, and its weighting schema allows for the generation of national estimates representing the vast majority of the U.S. population. NIS records include patient demographics, hospital characteristics, diagnoses, procedures, and comorbidities captured with International Classification of Diseases, Tenth Revision, Clinical Modification (ICD-10-CM) codes. Because the database is publicly available and contains only de-identified information, the present analysis was exempt from institutional review board oversight.

For this analysis, we included all adult (≥18 years) hospitalizations between 1 January and 31 December 2019, with a primary discharge diagnosis of obstructive sleep apnea (OSA), identified using ICD-10-CM code G47.33. We excluded encounters with missing data on age, sex, race, length of stay, hospitalization cost, or in-hospital mortality. Among these OSA admissions, secondary pulmonary hypertension (SPH) was identified using ICD-10-CM code I27.21. We then evaluated temporal trends and distributions for patient demographics, admission type, primary expected payer (Medicare, Medicaid, and private insurers including health maintenance organizations), neighborhood median income quartile, and hospital characteristics including location and teaching status (rural, urban non-teaching, and urban teaching). Pre-existing comorbidities, all-cause in-hospital mortality, major adverse cardiovascular events, and discharge disposition (routine home discharge, transfer to short-term rehabilitation, Skilled Nursing Facility, Intermediate Care Facility, home-health services, or discharge against medical advice) were also assessed. The primary outcome of this study was all-cause in-hospital mortality among obstructive sleep apnea (OSA) hospitalizations with secondary pulmonary hypertension (SPH). Secondary outcomes included (1) SPH-related hospitalization status among adults hospitalized with OSA, used to identify independent predictors of SPH, and (2) in-hospital outcomes and resource utilization comparing OSA patients with and without SPH, including all-cause mortality, major adverse cardiovascular and cerebrovascular events (MACCEs), length of stay, total hospitalization charges, and discharge disposition.

Statistical analyses incorporated NIS-provided discharge weights, strata, and clustering to account for the complex survey design and generate nationally representative estimates. Categorical variables were compared using weighted χ^2^ tests, and continuous variables were compared using Student’s *t*-test, as appropriate. Unadjusted analyses were performed to compare in-hospital outcomes and resource utilization between obstructive sleep apnea (OSA) hospitalizations with and without secondary pulmonary hypertension (SPH), including all-cause in-hospital mortality, major adverse cardiovascular and cerebrovascular events (MACCEs), length of stay, total hospitalization charges, and discharge disposition. Multivariable logistic regression analyses were subsequently conducted to identify independent predictors of (1) SPH-related hospitalization among OSA admissions and (2) all-cause in-hospital mortality among OSA patients with SPH. The objective was primarily exploratory, to identify predictors of secondary pulmonary hypertension-related hospitalizations and mortality using a large administrative dataset. As this study was exploratory and not hypothesis-driven, Bonferroni adjustment was not applied. A two-sided value of *p* < 0.05 was set for statistical significance in all analyses. IBM SPSS Statistics 22.0 (IBM Corp., Armonk, NY, USA) software was used to execute all statistical analyses.

## 3. Results

Of the total adult hospitalizations in 2019 with OSA (N = 2,317,136, median age of 66 [56–74] years, and males: 57.2%), 218,795 (9.4%) patients had SPH. The SPH cohort consisted more of older patients (70 years vs. 65 years), females (51% vs. 41.9%), Blacks (20.9% vs. 14.0%), patients with lower household income (29.7% vs. 27.6%), Medicare-insured (73.4% vs. 60.6%), and non-elective admissions (89.2% vs. 74.4%) when compared to the non-SPH cohort.

The SPH cohort also had a higher burden of complicated HTN (52.9% vs. 36.3%), DM with chronic complications (42.7% vs. 32.4%), hyperlipidemia (59.4% vs. 57.6%), obesity (53.7% vs. 50.6%), peripheral vascular disease (10.6% vs. 7.8%), chronic pulmonary disease (52.5% vs. 36.9%), history of prior MI (11.4% vs. 9.6%), stroke (9.0% vs. 8.6%) and venous thromboembolism (10.4% vs. 8.4%) (*p* < 0.001). However, uncomplicated hypertension (39.3% vs. 12.4%) and tobacco use disorder (13.5% vs. 10.9%) were more in the non-SPH cohort (*p* < 0.001). (Table 1: baseline characteristics).

Blacks (OR 1.63; 95%CI 1.57–1.70), Asian–Pacific Islanders (OR 1.17; 95%CI 1.05–1.30), admission from urban teaching hospitals (OR 1.12; 95%CI 1.04–1.21), hypertension (OR 1.45; 95%CI 1.41–1.48), diabetes with chronic complications (OR 1.11; 95%CI 1.09–1.14), obesity (OR 1.24; 95%CI 1.20–1.27), prior MI (OR 1.08; 95%CI 1.04–1.12), drug abuse (OR 1.28; 95%CI 1.17–1.39), chronic pulmonary disease (OR 1.60; 95%CI 1.56–1.64), thyroid disorders (OR 1.17; 95%CI 1.08–1.27), and prior VTE (OR 1.13; 95%CI 1.09–1.17) (*p* < 0.001) were all positive predictors of SPH, whereas females (OR 0.75; 95%CI 0.73–0.77), diabetics without chronic complications (OR 0.85; 95%CI 0.82–0.88), hyperlipidemia (OR 0.92; 95%CI 0.90–0.95), prior CABG (OR 0.88; 95%CI 0.84–0.92), stroke or TIA (OR 0.86; 95%CI 0.82–0.89), tobacco use disorder (OR 0.83; 95%CI 0.80–0.86), opioid use (OR 0.70; 95%CI 0.64–0.77), cannabis use (OR 0.87; 95%CI 0.78–0.97, *p* = 0.014), metastatic and malignant solid tumor cancers without metastasis (OR 0.61; 95%CI 0.56–0.66) and depression (OR 0.86; 95%CI 0.84–0.89) were negative predictors of SPH in obese patients (*p* < 0.001) (Table 2: SPH predictors). All regression results presented in Table 2 and Table 3 represent multivariable logistic regression models adjusted for sociodemographic characteristics, hospital factors, and clinically relevant comorbidities.

Odds of all-cause mortality were higher in hypertensives (OR 1.43; 95%CI 1.27–1.61), metastatic cancer (OR 2.73; 95%CI 2.04–3.65), and solid non-metastatic tumors (OR 1.65; 95%CI 1.26–2.15) (*p* < 0.001). Mortality was observed to be lower in diabetics without chronic complications (OR 0.78; 95%CI 0.63–0.96, *p* = 0.019), hyperlipidemia (OR 0.70; 95%CI 0.62–0.78, *p* < 0.001), obesity (OR 0.74; 95%CI 0.66–0.84. *p* < 0.001), prior TIA or stroke (OR 0.62; 95%CI 0.49–0.79, *p* < 0.001), tobacco use disorder (OR 0.77; 95%CI 0.62–0.97, *p* = 0.024), and prior VTE (OR 0.69; 95%CI 0.56–0.85, *p* < 0.001) (Table 3: ACM predictors).

**Figure 1:**
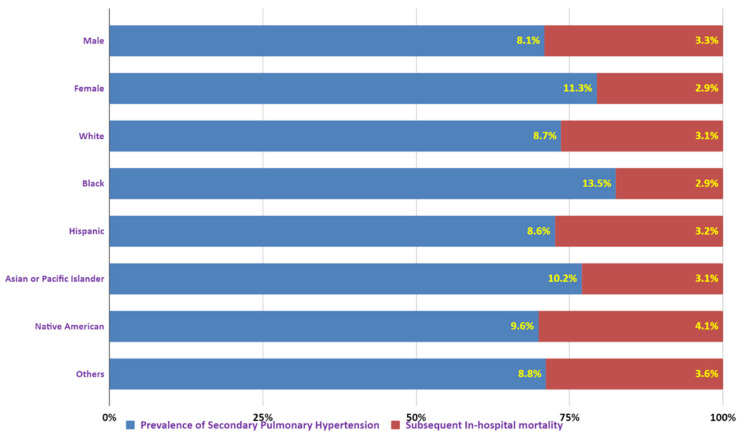
Prevalence of secondary pulmonary hypertension in obstructive sleep apnea by sex and race.

Overall prevalence of SPH was higher in females compared to males (11.3% vs. 8.1%); however, mortality was higher in males compared to females (3.3% vs. 2.9%). Similarly, prevalence was higher in Blacks compared to Whites (13.5 vs. 8.7), Hispanics (8.6%), and Asian–Pacific Islanders (10.2%). However, mortality was, least in Blacks (2.9%) compared to other ethnic groups (Figure 1).

## 4. Discussion

The present study utilized a nationally representative sample of hospitalized obstructive sleep apnea (OSA) patients from the National Inpatient Sample in 2019 to investigate the predictors of secondary pulmonary hypertension (SPH)-related hospitalizations and subsequent mortality. Our findings indicate the following: 1. The prevalence of SPH is higher in females, but males have higher in-hospital mortality rates. 2. Black ethnic groups had higher prevalence of SPH, but other races, such as Hispanics, Asian–Pacific Islanders, Whites, and Native Americans, also had higher in-hospital mortality rates. 3. Several risk factors were identified as predictive of SPH or in-hospital mortality. Hyperlipidemia, uncomplicated diabetes, obesity, prior TIA or stroke, tobacco use disorder, and prior VTE were protective risk factors, while hypertension, metastatic cancer, solid non-metastatic tumors were associated with higher in-hospital all-cause mortality. 4. Positive predictors of SPH included admission from urban teaching hospitals, hypertension, diabetes with chronic complications, obesity, prior MI, drug abuse, chronic pulmonary disease, thyroid disorders, and prior VTE, while negative predictors were females, diabetics without chronic complications, hyperlipidemia, prior CABG, stroke or TIA, tobacco use disorder, opioid use, cannabis use, metastatic and malignant solid tumor cancers without metastasis, and depression in OSA patients.

Gender disparities have been mentioned previously in various studies, where the prevalence of SPH is higher in females but mortality remains higher in the male counterparts [9]. Our finding that females have a higher prevalence of SPH is consistent with previous studies, such as the Registry to Evaluate Early and Long-term Pulmonary Arterial Hypertension Disease Management (REVEAL Registry) for characteristics of patients with pulmonary arterial hypertension [10]. The higher prevalence of pulmonary artery disease in females may be attributed to hormonal influences [11,12]. Estrogen and its similar receptors are synthesized locally by the pulmonary arterial smooth muscle cells, leading to local proliferation of smooth muscles. This might explain the higher prevalence of this disease in females. However, it must also be noted that estrogen has shown a protective effect in several animal trials, given its effect in slowing down right ventricular hypertrophy and antiproliferative effects in pulmonary artery smooth muscle cells [13,14]. On the contrary, low levels of estrogen in men explain the lower prevalence; however, absence of estrogen also fails to offset the protective effects of the same in males. This might explain why males had poor outcomes.

Beyond hormonal influences, recent studies have highlighted anatomical and physiological differences that may explain the higher prevalence of SPH in women. A recent study demonstrated that women have a smaller total pulmonary vascular volume (TVV) and a higher proportion of small pulmonary vessels (cross-sectional area < 5 mm^2^), even after adjusting for lung size, suggesting increased susceptibility to pulmonary vascular resistance under stress [15]. Furthermore, Zamanian et al. reported that women exhibit higher pulmonary vascular resistance (PVR), lower pulmonary artery compliance (PAC), and reduced cardiac output (CO) compared to men, hemodynamic changes that increase the load on the right ventricle in conditions such as OSA-related SPH [16]. Interestingly, despite these less-favorable hemodynamic parameters, Ma et al. found that women may demonstrate a more adaptive right ventricular response, with a higher pulmonary artery pulsatility index (PAPi), right ventricular stroke work index (RVSWI) [17], and a lower right atrial to pulmonary capillary wedge pressure (RA:PCWP) ratio, which may partially explain their relatively better in-hospital outcomes [18]. These findings underscore the need to consider sex-specific anatomical and physiological traits when evaluating SPH risk in OSA patients.

Right ventricular remodeling and mass are maladaptive in patients with pulmonary hypertension and have been proven to be predictors of mortality and poor outcomes in patients with pulmonary arterial hypertension [19]. Obesity is associated with increased pulmonary arterial hypertension in patients with OSA, which is why obese individuals should be screened for underlying sleep-related disorders. In our analysis, obesity was a positive predictor of SPH; however, it also negatively influenced in-hospital mortality outcomes in the SPH cohort, as many studies have implicated the obesity paradox of in-hospital mortality, which is especially driven by critically ill patients [20]. Nonetheless, using an administrative database cannot eliminate these confounders as to who was critically ill and who was not.

Our analysis also revealed that cardiovascular risk factors, such as hypertension and diabetes, are associated with endothelial dysfunction, which is relevant, as these risk factors were positive predictors of SPH along with evidence of coronary artery disease, such as prior history of MI and coronary revascularization. Chronic pulmonary disease encompassing major diseases like chronic obstructive pulmonary disease, asthma, and emphysema, as well as rare diseases like pulmonary fibrosis, lymphangioleiomyomatosis, sarcoidosis, and pulmonary Langerhans cell histiocytosis, are also associated with SPH and fall under groups 3 and 5 of the clinical classification system of pulmonary hypertension, which are secondary causes of pulmonary hypertension [21].

Venous thromboembolism has a recurrence rate of 5–7% per year, and it is more than 50 times higher than in patients without previous VTE [22]. In our study, prior VTE was associated with developing SPH. This can be explained by chronic thromboembolic pulmonary hypertension, which has an incidence rate in VTE patients with 3.5 per 1000 person-years [23].

Finally, our study sheds light on the racial and ethnic disparities in the prevalence and outcomes of SPH-related hospitalizations. Racial disparities have also been previously described with elevated pulmonary artery pressures [1]. In a large national PAH registry, racial and ethnic distribution of pulmonary hypertension was explored [24]. In our study, while Blacks had a higher prevalence of SPH, they had better outcomes compared to other racial and ethnic groups. Contrary to our findings, in this small retrospective cohort study, minority groups were associated with worse outcomes [25]. Greater prevalence for secondary causes of PH can be attributed to higher prevalence of autoimmune diseases such as scleroderma, rheumatoid arthritis [26], HIV infection [27], and OSA in Black individuals [28]. The reasons behind these disparities are complex and multifactorial, and further studies are needed to identify the underlying mechanisms and develop targeted interventions to address them. Moreover, patients with OSA complicated by SPH had a significantly longer median hospital stay (5 vs. 3 days, *p* < 0.001) and incurred higher median total hospital charges ($46,821 vs. $42,908, *p* < 0.001), indicating a substantial increase in resource utilization and economic burden. These findings highlight the importance of early identification and management of SPH in OSA patients to help mitigate hospitalization costs and reduce the length of stay.

## 5. Limitations

A few limitations of our study: 1. The study relied on administrative data from the NIS database, which has inherent limitations such as coding errors and missing data, so we could not distinguish among specific pulmonary hypertension subtypes (for example, chronic thromboembolic pulmonary hypertension, left heart disease-related PH, or lung disease-related PH), nor could we assess hemodynamic severity or functional class. 2. Comparisons between obstructive sleep apnea patients with and without SPH were descriptive and unadjusted, and should be interpreted cautiously given the severity of PH and the lack of matching on key clinical factors. 3. This study used a single cross-sectional NIS sample from 2019, which may limit generalizability to other time periods and prevent assessment of longitudinal disease progression. The dataset also lacked information on OSA severity (such as the apnea–hypopnea index and nocturnal hypoxemia burden), adherence to positive airway pressure therapy, and overlap syndromes (for example, obesity hypoventilation), all of which may influence the development and prognosis of SPH. 4. Despite multivariable adjustment, residual confounding from unmeasured factors (for example, disease duration, medications, right ventricular function, socioeconomic status, and access to care) remains likely; therefore, the associations observed should not be interpreted as causal. 5. Finally, although the study identifies demographic and clinical predictors of SPH-related hospitalization and in-hospital mortality, these findings need further validation via prospective studies with detailed hemodynamic data, standardized functional class, and long-term follow-up.

## 6. Conclusions

In conclusion, our study provides insights into the predictors of SPH-related hospitalizations and subsequent mortality in adults with obstructive sleep apnea. Our findings highlight the significant impact of gender, race, cardiovascular risk factors, and prior medical conditions on the development of SPH and in-hospital mortality outcomes. Our study also highlights the importance of screening for underlying sleep-related disorders in obese individuals, as obesity was found to be a positive predictor of SPH and influenced in-hospital mortality outcomes in the SPH cohort. Furthermore, the association between prior VTE and SPH underscores the need for close monitoring and management of patients with a history of VTE. Overall, our study sheds light on the complex interplay of various risk factors in the development and outcomes of SPH in adults with obstructive sleep apnea. Future studies are needed to confirm these findings and further investigate potential interventions to improve outcomes in this population. Clinicians should be aware of these predictors to identify patients at higher risk of developing SPH and to provide timely and appropriate interventions.

## Figures and Tables

**Table 1:** Baseline characteristics in obstructive sleep apnea patients with vs. without secondary pulmonary hypertension.

Variables		Secondary Pulmonary Hypertension				
		NO		YES		
		n	Percentage %	n	Percentage %	*p*-Value
Demographics						
Age (years) at admission, median [IQR]	18–44	181,100	8.6%	9935	4.5%	<0.001
	45–64	810,880	38.6%	65,450	29.9%	
	≥65	1,106,360	52.7%	143,410	65.5%	
Sex	Male	1,218,650	58.1%	107,285	49.0%	<0.001
	Female	879,545	41.9%	111,505	51.0%	
Race	White	1,563,920	76.3%	149,805	70.0%	<0.001
	Black	286,550	14.0%	44,795	20.9%	
	Hispanic	126,725	6.2%	11,900	5.6%	
	Asian/Pacific Islanders	25,830	1.3%	2940	1.4%	
	Native Americans	11,375	0.6%	1210	0.6%	
	Others	34,545	1.7%	3350	1.6%	
Median household income	0–25th	570,835	27.6%	64,105	29.7%	<0.001
	26–50th	547,855	26.5%	56,965	26.4%	
	51–75th	539,590	26.1%	54,845	25.4%	
	76–100th	411,330	19.9%	40,090	18.6%	
Primary expected payer	Medicare	1,270,280	60.6%	160,440	73.4%	<0.001
	Medicaid	199,450	9.5%	19,450	8.9%	
	Private with HMO	524,585	25.0%	31,600	14.5%	
	Self-pay	38,715	1.8%	2950	1.3%	
	No charges	2530	0.1%	150	0.1%	
	Others	60,550	2.9%	4040	1.8%	
Type of admission	Non-elective	1,559,150	74.4%	195,040	89.2%	<0.001
	Elective	537,430	25.6%	23,585	10.8%	
Location of hospital	Rural	162,310	7.7%	16,120	7.4%	<0.001
	Urban non-teaching	363,300	17.3%	34,790	15.9%	
	Urban teaching	1,572,730	75.0%	167,885	76.7%	
Comorbidities						
Complicated hypertension		762,305	36.3%	115,810	52.9%	<0.001
Uncomplicated hypertension		823,850	39.3%	27,065	12.4%	<0.001
						
Diabetes with chronic complications		679,560	32.4%	93,405	42.7%	<0.001
Diabetes without chronic complications		331,225	15.8%	25,860	11.8%	<0.001
Hyperlipidemia		1,209,365	57.6%	129,890	59.4%	<0.001
Obesity		1,061,230	50.6%	117,410	53.7%	<0.001
Tobacco use disorder		282,455	13.5%	23,830	10.9%	<0.001
Peripheral vascular disease		164,530	7.8%	23,300	10.6%	<0.001
Prior myocardial infarction		201,150	9.6%	24,840	11.4%	<0.001
Prior PCI		16,370	0.8%	2005	0.9%	<0.001
Prior CABG		198,465	9.5%	21,130	9.7%	0.002
Drug abuse		63,300	3.0%	6515	3.0%	0.31
Chronic pulmonary disease		774,210	36.9%	114,835	52.5%	<0.001
Prior TIA and stroke		180,655	8.6%	19,755	9.0%	<0.001
Prior VTE		176,260	8.4%	22,680	10.4%	<0.001
In-hospital outcomes						
All-cause mortality		33,170	1.6%	6805	3.1%	<0.001
MACCE		195,250	9.3%	24,470	11.2%	<0.001
Disposition	Routine	1,223,660	58.3%	103,485	47.3%	<0.001
	Short-term hospital	41,670	2.0%	5495	2.5%	
	Others *	365,375	17.4%	47,770	21.8%	
	Home-healthcare	416,445	19.8%	53,535	24.5%	
	AMA	17,595	0.8%	1645	0.8%	
Length of stay (days), median [IQR]		3		5		<0.001
Total charges (USD), median [IQR]		42,908		46,821		<0.001

IQR—inter-quartile range; HMO—health maintenance organization; PCI—percutaneous coronary intervention; CABG—coronary artery bypass grafting; TIA—transient ischemic stroke; VTE—venous thromboembolism; MACCEs—major adverse cardiovascular and cerebrovascular events; AMA—against medical advice. * Includes Skilled Nursing Facility (SNF), Intermediate Care Facility (ICF), and another type of facility. *p* < 0.05 was considered significant.

**Table 2:** Independent predictors of secondary pulmonary hypertension-related hospitalizations among adults with obstructive sleep apnea derived from multivariable logistic regression analysis.

Predictors of Secondary Pulmonary Hypertension		Odds Ratio	95% Confidence Interval		*p*-Value
			Lower	Upper	
Sex	Male vs. Female	0.75	0.73	0.77	<0.001
Race	Black vs. White	1.63	1.57	1.70	<0.001
	Hispanic vs. White	1.06	1.00	1.13	
	API vs. White	1.17	1.05	1.30	
	Native American vs. White	1.17	1.01	1.36	
	Others vs. White	1.12	1.02	1.22	
Location of hospital	Urban non-teaching vs. Rural	0.98	0.90	1.07	<0.001
	Urban teaching vs. Rural	1.12	1.04	1.21	
Hypertension	Yes vs. No	1.45	1.41	1.48	<0.001
Diabetes with chronic complications	Yes vs. No	1.11	1.09	1.14	<0.001
Diabetes without chronic complications	Yes vs. No	0.85	0.82	0.88	<0.001
Hyperlipidemia	Yes vs. No	0.92	0.90	0.95	<0.001
Obesity	Yes vs. No	1.24	1.20	1.27	<0.001
Prior myocardial infarction	Yes vs. No	1.08	1.04	1.12	<0.001
Prior CABG	Yes vs. No	0.88	0.84	0.92	<0.001
Prior TIA and stroke	Yes vs. No	0.86	0.82	0.89	<0.001
Tobacco use disorder	Yes vs. No	0.83	0.80	0.86	<0.001
Drug abuse	Yes vs. No	1.28	1.17	1.39	<0.001
Opioid related disorders	Yes vs. No	0.70	0.64	0.77	<0.001
Cannabis use disorder	Yes vs. No	0.87	0.78	0.97	0.014
Metastatic cancer	Yes vs. No	0.61	0.56	0.66	<0.001
Solid tumor without metastasis	Yes vs. No	0.80	0.75	0.85	<0.001
Depression	Yes vs. No	0.86	0.84	0.89	<0.001
Chronic pulmonary disease	Yes vs. No	1.60	1.56	1.64	<0.001
Other thyroid disorders	Yes vs. No	1.17	1.08	1.27	<0.001
Prior VTE	Yes vs. No	1.13	1.09	1.17	<0.001

Multivariate regression analysis was adjusted for sociodemographic characteristics like age, sex, race, household income, payer type, type of admission, hospital region, location of the hospital, and comorbidities like complicated hypertension, diabetes with and without complications, hyperlipidemia, obesity, prior myocardial infarction, prior PCI, prior CABG, prior TIA and stroke without neurologic deficit, alcohol use, tobacco use disorder, drug abuse, opioid related disorders, cannabis use disorder, metastatic cancer, solid organ cancer, depression, chronic lung diseases, thyroid diseases, and prior VTE. PCI—percutaneous coronary intervention; CABG—coronary artery bypass grafting; TIA—transient Ischemic stroke; VTE—venous thromboembolism.

**Table 3:** Independent predictors of all-cause in-hospital mortality among obstructive sleep apnea patients with secondary pulmonary hypertension derived from multivariable logistic regression analysis.

Predictors of All-Cause Mortality		Odds Ratio	95% Confidence Interval		*p*-Value
			Lower	Upper	
Sex	Male vs. Female	1.12	1.00	1.25	0.048
Location of hospital	Urban non-teaching vs. Rural	0.82	0.63	1.07	0.001
Hypertension	Yes vs. No	1.43	1.27	1.61	<0.001
Diabetes without chronic complications	Yes vs. No	0.78	0.63	0.96	0.019
Hyperlipidemia	Yes vs. No	0.70	0.62	0.78	<0.001
Obesity	Yes vs. No	0.74	0.66	0.84	<0.001
Prior TIA and stroke	Yes vs. No	0.62	0.49	0.79	<0.001
Tobacco use disorder	Yes vs. No	0.77	0.62	0.97	0.024
Metastatic cancer	Yes vs. No	2.73	2.04	3.65	<0.001
Solid tumor without metastasis	Yes vs. No	1.65	1.26	2.15	<0.001
Prior VTE	Yes vs. No	0.69	0.56	0.85	<0.001

Multivariate regression analysis was adjusted for sociodemographic characteristics like age, sex, race, household income, payer type, type of admission, hospital region, location of the hospital, and comorbidities like complicated hypertension, diabetes with and without complications, hyperlipidemia, obesity, prior myocardial infarction, prior PCI, prior CABG, prior TIA and stroke without neurologic deficit, alcohol use, tobacco use disorder, drug abuse, opioid related disorders, cannabis use disorder, metastatic cancer, solid organ cancer, depression, chronic lung diseases, thyroid diseases, and prior VTE. PCI—percutaneous coronary intervention; CABG—coronary artery bypass grafting; TIA—transient Ischemic stroke; VTE—venous thromboembolism.

## Data Availability

The data analyzed in this study are publicly available from the HCUP’s National Inpatient Sample. Access to the dataset is subject to HCUP data use agreements. Further inquiries can be directed to the corresponding author.

## Abbreviations

SPH (Secondary Pulmonary Hypertension); OSA (Obstructive Sleep Apnea); PH (Pulmonary Hypertension); mPAP (Mean Pulmonary Arterial Pressure); PAWP (Pulmonary Arterial Wedge Pressure); COPD (Chronic Obstructive Pulmonary Disease); MI (Myocardial Infarction); VTE (Venous Thromboembolism); TIA (Transient Ischemic Attack); PCI (Percutaneous Coronary Intervention); CABG (Coronary Artery Bypass Grafting); MACCEs (Major Adverse Cardiovascular and Cerebrovascular Events); CO (Cardiac Output); PAC (Pulmonary Artery Compliance); PVR (Pulmonary Vascular Resistance); PAPi (Pulmonary Artery Pulsatility Index); RVSWI (Right Ventricular Stroke Work Index); RA:PCWP (Right Atrial to Pulmonary Capillary Wedge Pressure Ratio); PAH (Pulmonary Arterial Hypertension); OHS (Obesity Hypoventilation Syndrome).
